# Edible Insects: Food Safety Challenges and Regulatory Perspectives

**DOI:** 10.3390/foods15112018

**Published:** 2026-06-04

**Authors:** Sara A. M. Silva, Vasco T. Esteves, Tiago Ribeiro, José Andrade, Cristina Couto, Joana C. Prata

**Affiliations:** 1 UCIBIO—Applied Molecular Biosciences Unit, Translational Toxicology Research Laboratory, University Institute of Health Sciences—CESPU (1H-TOXRUN, IUCS-CESPU), 4585-116 Gandra, Portugal; jose.andrade@iucs.cespu.pt (J.A.); joana.prata@iucs.cespu.pt (J.C.P.); 2 Associate Laboratory i4HB—Institute for Health and Bioeconomy, University Institute of Health Sciences—CESPU, 4585-116 Gandra, Portugal; 3 Tecmafoods, Rua Sousa Prata 678, 4465-757 Leça do Balio, Portugal; geral@tecmafoods.com (V.T.E.); transformacao@tecmafoods.com (T.R.); 4 UCIBIO—Research Unit on Applied Molecular Biosciences, Forensic Science Research Laboratory, University Institute of Health Sciences (1H-TOXRUN, IUCS-CESPU), 4585-116 Gandra, Portugal

**Keywords:** edible insects, insect-based food products, food safety, food contaminants

## Abstract

Edible insects have emerged as a promising alternative to conventional livestock as the global demand for sustainable protein sources rises. Ensuring the safety of insect-based foods is crucial for consumer acceptance and regulatory approval. This review provides a comprehensive overview of the primary chemical and microbiological contaminants associated with edible insects, including heavy metals, pesticides, veterinary drugs, persistent organic pollutants (POPs), mycotoxins, microbiological hazards, and allergenic risks. Current evidence indicates that, when insects are farmed and processed under controlled conditions and in compliance with existing European Union regulations, contaminant levels are generally low and within the range of those found in traditional animal-derived foods. Most studies report that current risks are primarily linked to substrate quality and storage practices. Allergenic risks, particularly cross-reactivity with crustacean and mite allergens, remain a crucial consideration for individuals with sensitivities. Despite these reassuring findings, knowledge gaps persist regarding insect-specific contaminant limits, the metabolic fate of toxins, and the long-term safety of consuming novel insect-derived products. Continued research, targeted monitoring, and regulatory adaptation will be essential to ensure the safe and sustainable integration of insect-based foods into the human diet.

## 1. Introduction

The global interest in insect-based food products has surged in recent years, driven by a growing recognition of their nutritional value and sustainability benefits, but above all, the challenge of feeding an estimated 9.1 billion people by 2050 [1,2]. This demand has prompted research and development to seek alternative production systems and sustainable food sources (insects, fungi, cultured meat, micro and macro algae) to fulfilll the United Nations Sustainable Development Goals regarding nutrition and well-being.

Insects offer a rich source of high-quality protein, essential amino acids, healthy fats, fiber, and various vitamins and minerals—including iron, zinc, calcium, and vitamin B12—often in amounts comparable to or exceeding those found in conventional animal- and plant-based foods [3,4]. Additionally, edible insects contain bioactive compounds (e.g., chitin, polyunsaturated fatty acids (PUFAs), peptides, and antioxidants) that may promote health benefits such as enhanced antioxidant, antimicrobial, and immunomodulatory effects, improved immune function, and a decreased risk of chronic diseases [5].

**Figure 1:**
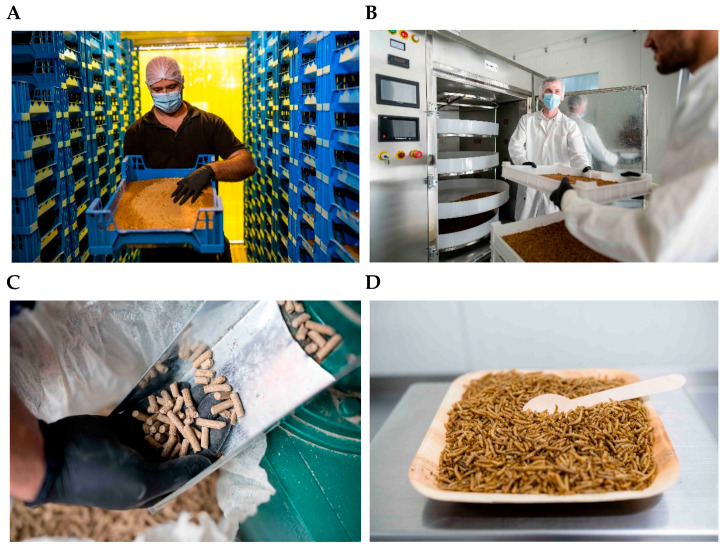
Stages of edible insect production and processing. (**A**) Handling and storage of insect substrate in stackable trays under controlled production conditions. (**B**) Processing and drying operations during industrial-scale insect production. (**C**) Pelletized insect-derived frass. (**D**) Whole-insect product (Courtesy of Tecmafoods).

Beyond their nutritional merits, insects are increasingly considered sustainable food sources. Insect farming requires significantly less land and water and has a higher feed-to-protein conversion efficiency compared to traditional livestock [6]. For example, crickets have a feed conversion ratio of approximately 2.1 kg of feed per 1 kg of edible weight gained, whereas poultry require about 4.5 kg, pigs about 9.1 kg, and cattle up to 25 kg [1,2]. Insect farming produces significantly lower greenhouse gas (GHG) emissions (e.g., pigs generate 10 to 100 times more GHG per kg of weight than mealworms) and can be integrated into circular economy models by using organic food waste as feed, thus reducing environmental contamination and closing nutrient loops [6]. Moreover, byproducts of insect production can be integrated into the circular economy. For instance, chitin and chitosan have biotechnological applications, while insect frass can be used as an organic fertilizer [7,8]. The current market for insect-based food products is diverse and rapidly evolving. Products available range from whole insects (highest market share), such as roasted crickets, mealworms, and grasshoppers, to processed forms including insect powders, flours, and protein concentrates, which are increasingly used as ingredients in a variety of foods (Figure 1) [9]. These ingredients are incorporated into snacks, protein bars, pasta, baked goods, and even meat analogs such as burgers, offering consumers a familiar format with the nutritional benefits of insect protein [10]. As consumer acceptance grows and food innovation continues, the range of insect-based products is expected to expand, with functional foods, paleo diet-specific products, and meat alternatives projected to see high growth rates in the coming years [9].

As insect-based foods become more widely produced and consumed, ensuring their safety and quality is crucial. Contaminants, whether chemical, microbiological, or physical, pose potential risks to consumer health and can undermine confidence in these novel food products. Identifying and managing these contaminants is essential not only for protecting public health but also for supporting the continued growth and acceptance of the edible insect sector.

The current scoping review aims to present an updated overview of the potential contaminants present in insect protein-based food products, including chemical and biological contaminants, and allergenic risks, highlighting the primary sources of contamination that may occur throughout the production chain. Furthermore, the review aims to discuss the current regulatory EU framework applicable to these products and to identify the main regulatory gaps and challenges that persist. The focus on the European Union regulatory framework is particularly relevant due to the significant recent activity in this area, including updated legislation specifically targeting novel foods such as edible insects. Moreover, the projected rapid growth of the edible insect sector within the EU economy underscores the urgency of regulatory adaptation. The insect protein market in Europe alone is expected to grow from USD 192.73 million in 2025 to USD 3.72 billion by 2033, exhibiting a remarkable compound annual growth rate of approximately 45% [11]. This dynamic regulatory and economic context underpins the importance of examining the EU’s role in shaping the safe and sustainable future of insect-based food products.

## 2. Materials and Methods

This review sourced peer-reviewed articles from the Web of Science, Google Scholar, and Scopus databases, focusing on publications in English released between 1 January 2019 and 1 June 2025. For contaminant groups with limited data available, the search was expanded to include relevant studies published before this period to ensure a comprehensive summary. The literature search employed combinations of the following keywords: “edible insects,” “insect-based food,” “food safety,” “contaminants,” “heavy metals,” “pesticide residues,” “veterinary drugs,” “persistent organic pollutants,” “mycotoxins,” “microbiological contamination,” and “allergens”. Studies were excluded if they did not provide relevant contaminant data. After screening, 72 studies met the inclusion criteria and were analyzed.

## 3. Status of the Edible Insect Industry

Edible insects are consumed by approximately 2 billion people worldwide and have long been a staple in many traditional dietary patterns, namely those in Asia, Africa, and South America [4]. However, they are not yet included in consumers’ regular diets and animal feeds in Europe and North America [12]. A recent study estimated that approximately 2205 species of insects are consumed in about 128 countries globally. Asia leads in diversity, with 932 edible insect species, followed by North America (including Mexico) with 529, Africa with 464, and South America with 300 species [13]. The global distribution of edible insect species per group is presented in Table 1.

In the European Union (EU), insects have been classified as “novel foods” since 2015 and must be approved by the European Commission (EC) before they can be marketed [14]. Currently, four insect species have been authorized for human consumption: the yellow mealworm (*Tenebrio molitor* larva; Regulation (EU) 2021/882; 2022/169), migratory locusts (*Locusta migratoria*; Regulation (EU) 2021/1975), house cricket (*Acheta domesticus*; Regulation (EU) 2022/188; 2023/5), and lesser mealworm (*Alphitobius diaperinus* larva; Regulation (EU) 2023/58). In the United States (US), insect-based products are not subject to insect-specific food regulations but must comply with general food safety standards established by the Food and Drug Administration (FDA) under the Federal Food, Drug, and Cosmetic Act [15]. Similarly, Mexico, Australia and New Zealand have no independent legal framework or specific government legislation for insect-based products [16]. In Canada, regulations stipulate that if a food item has a documented history of traditional consumption elsewhere in the world, it can be sold here without further regulatory constraints but must first undergo a novelty determination process [15].

Despite the existing authorizations under the EU’s Novel Food regulation, insect-based food producers still face practical challenges in industrial application. The approval process can be lengthy and costly, limiting market entry, especially for smaller enterprises. Moreover, rigorous traceability requirements for substrates used in insect farming and the absence of insect-specific maximum residue limits (MRLs) further complicate quality control and risk management, as producers must rely on extrapolated standards from other animal-origin foods. These factors collectively pose bottlenecks for rapid industrial scaling and innovation in the edible insect sector.

## 4. Current Regulatory Framework for Edible Insects as Food in the European Union

The foundation of EU food law as it applies to insects was laid by Regulation (EC) No 258/97 and Regulation (EC) No 1852/2001, adopted in 1997, which defined as “novel” any food or food ingredient not used for human consumption to a significant degree before 15 May 1997 [17,18]. However, this regulation created significant ambiguity when applied to insects, and several Member States differed on whether whole insects fell within the scope of the legislation, leading to diverging national interpretations of the legal status of these foods and ingredients [19].

The legal gap in the status of edible insects was only filled in 2015, with the adoption and update of the Novel Food Regulation, Regulation (EU) 2015/2283, applicable since 1 January 2018 [14]. The expanded definition of novel food explicitly includes “food consisting of, isolated from or produced from animals or their parts” in the categories of food that may constitute a novel food. It further states that “those categories should cover whole insects and their parts” [14]. The revised framework introduced several important changes compared with the previous legislation. First, it established a centralized authorization process, replacing the earlier Member State-based system. Second, it created the Union List of Authorized Novel Foods, allowing authorized insect products to be marketed throughout the EU under specified conditions of use and labeling requirements (mandatory allergen information and species-specific product). Third, the regulation introduced a simplified notification procedure for traditional foods from third countries with a documented history of safe consumption. Unlike the standard authorization pathway, this route places the burden of demonstrating safe use on the applicant, with the European Food Safety Authority (EFSA) and Member States playing a reactive role, as further detailed in Commission Implementing Regulation (EU) 2017/2468 [20]. In the context of edible insects, however, this procedure has seen limited practical use, as demonstrating a sufficiently documented history of safe consumption to satisfy EFSA’s scientific standards remains a considerable evidentiary challenge. Finally, the updated legislation strengthened efficiency and transparency and promoted innovation through data protection provisions for applicants. Under this regulation, market authorization is granted following the submission of an application to the EC, the safety evaluation of the novel food by the EFSA, and a favorable vote by EU Member States.

The first authorizations began materializing from 2021 onwards. Dried *T. molitor* larva was authorized in June 2021, followed by frozen, dried and powder forms of *L. migratoria* in November 2021, and frozen, dried and powder forms of *A. domesticus* in February 2022. Since then, the number of authorized insect-based novel foods and safety opinions issued by the EFSA has continued to increase. As of May 2026, EFSA has published at least 10 generally positive safety opinions on insect-based Novel Food applications under Regulation (EU) 2015/2283. These opinions generally concluded that the products were safe under the proposed conditions of use, while highlighting allergenicity as a relevant risk factor. These opinions cover several insect species and product formulations in forms such as whole insects, powders, frozen products, and UV-treated preparations [21]. In parallel with novel food legislation, edible insect production in Europe must also comply with broader food safety and hygiene legislation, including Regulation (EC) No 178/2002 (General Food Law) [22] and Regulation (EC) No 852/2004 on food hygiene [23]. Specific hygiene rules for insects intended for human consumption have also been considered through proposed amendments to Regulation (EC) No 853/2004 [24], aiming to establish dedicated hygiene requirements for insect production and processing [25]. These proposals reinforce existing restrictions on rearing substrates established under Regulations (EC) No 1069/2009 [26] and No 142/2011 [27], which prohibit the use of manure, catering waste, and other waste materials, while limiting insect feed to substrates of vegetable origin or specifically authorized animal-derived materials [25].

## 5. Chemical Contaminants

Chemical contaminants represent a crucial aspect of food safety in insect-based products, as insects are capable of accumulating pollutants from their environment. Bioaccumulation depends on the chemical, insect species, and life stage, as well as the particular substrate and rearing conditions [28]. The main categories of chemical contaminants identified in edible insects include heavy metals, pesticides, persistent organic pollutants (POPs), veterinary drugs, and plant toxins.

### 5.1. Heavy Metals and Metalloids

Heavy metals, including mercury (Hg), lead (Pb), cadmium (Cd), and chromium (Cr)(VI), are among the most significant chemical contaminants of concern due to their high toxicity and potential for bioaccumulation [29]. Indeed, the accumulation of heavy metals in edible insects is a significant concern, given their capacity to store these metals in their bodies [30]. Zhang et al. [31] demonstrated the bioaccumulation of Hg, Pb and Cd along the food chain, from heavy metal-contaminated soil to plants, then from plants to herbivore insects, and finally from herbivore insects to carnivore insects.

This bioaccumulation can occur through several pathways. Environmental exposure is a significant factor, as insects farmed in polluted areas may absorb these metals from contaminated water, soil, or air. Muhammad et al. [32] demonstrated that insects such as fingerlings (*Oreochromis niloticus*), grasshoppers (*Zonocerus variegatus*), locusts (*Schistocerca gregaria*), and termites (*Macrotermes bellicosus*) collected near agricultural areas contaminated with poultry manure and agricultural runoff exhibited significantly higher concentrations of metals. In 2019, Kasozi et al. [33] studied *Ruspolia nitidula* grasshoppers harvested in rural and urban areas of Uganda. Results show that grasshoppers in central Uganda were significantly contaminated with Pb, which was attributed to the abundance of forages close to heavy industrial discharges during the swarming seasons of these grasshoppers.

Additionally, the composition of insect feed plays a crucial role; insects are often fed agricultural by-products, organic waste, or specially formulated diets, all of which can contain trace amounts of heavy metals that are readily transferred and accumulated depending on the insect species and the type of metal present. Kofroňová et al. [34] showed that black soldier fly, *Hermetia illucens*, larvae fed on sewage sludge exceeded the limits for heavy metals, particularly Cd and Pb, even though they experienced enhanced larval growth. Furthermore, a study involving *Tenebrio molitor* L. larvae revealed a statistically significant correlation between the Hg content in feeding substrates and the larvae of *T. molitor*, which is known to bioaccumulate in insects. It was suggested that the high levels were due to organic carrots, which are generally rich in Pb and supplied as a water source for larvae [35].

Finally, processing methods, including drying, grinding, and packaging, can introduce further contamination if the equipment used or additives introduced during these stages are not properly controlled. Currently, the EU has only established limits for Pb and Cd (Table 2), even though a few studies have highlighted the presence of these and other toxic metals in commercially available insect-based products intended for human consumption, with concentrations varying according to the insect species, product type, and geographical origin.

Kolakowski et al. [36] analyzed cricket- and silkworm-based products, finding arsenic (As) levels ranging from 0.030 to 0.34 mg/kg, Cd from 0.031 to 0.23 mg/kg, Pb from 0.019 to 0.059 mg/kg, and Hg from 0.00094 to 0.028 mg/kg. Poma et al. [37] investigated a broader spectrum of metals, including As, Cd, cobalt (Co), Cr, copper (Cu), nickel (Ni), Pb, tin (Sn), and zinc (Zn), in various edible insects (greater wax moth, migratory locust, mealworm beetle, buffalo worm) and commercial insect-based foods (worm-based “bugballs” and “bugburger” and cricket croquettes) currently commercialized in Belgium. They confirmed the presence of these elements but noted variability between products and species. Kosečková et al. [38] assessed 14 minerals in cricket powders and popular edible insect species in the Czech Republic, reporting maximum Cd and Pb contents of 0.014 and 0.019 mg/100 g, respectively, which are relatively low compared to the EU limits (Table 2). Sikora et al. [39] examined 14 insect-based products from EU online stores, finding median levels of Al, As, Cd, Ni, and Pb of 0.028, 0.0026, 0.0278, and 0.016 mg/100 g, respectively. Cd and As were consistently below EFSA limits (no limits for insect-based food), while Hg levels were below the method detection limit (<0.015 mg/100 g) in all products. The highest contents of Al, As, Cd, and Pb were observed in a product containing *T. molitor*, and one based on *A. diaperinus* contained the highest content of Ni.

Overall, while heavy metals/metalloids such as As, Cd, Pb, and Hg are present in insect-based foods, most studies indicate that their concentrations are generally within established safety limits set for other types of food products; however, some products, especially those with high insect content (e.g., 100% insect powder), may occasionally exceed regulatory thresholds for certain metals, like Pb. As there are no specific regulatory limits for other heavy metals aside from Pb and Cd for insect-based products, concentrations are usually evaluated against the reference values and risk assessments provided by the EFSA for other food products (e.g., meat, food supplements, rice-based products, cereals and cereal products).

### 5.2. Pesticides and Veterinary Drugs

Pesticide and veterinary drug residues, which are bioactive substances of anthropogenic sources, are important chemical contaminants to consider in insect-based foods, as insects may be exposed to these substances through their feed, rearing environment, or during processing [28]. The presence of such residues raises food safety concerns, particularly regarding the potential for bioaccumulation and the risk of consumer exposure to pharmacologically active substances.

Pesticide residues can contaminate insects when they are reared on plant-based substrates that have been treated with these chemicals [12]. A study involving 511 pesticides across 47 insect samples found that most samples contained pesticide residues below the Canadian maximum residue limits defined for food [36]. Most samples (64%) contained only a single pesticide residue, while eight samples showed no detectable pesticide residues at all. Only a small number showed two or more residues, and very few exceeded compliance levels. Overall, seven pesticides (glyphosate, chlorfenapyr, chlorpyrifos, ethoxyquin, trifloxystrobin, tris(chloropropyl) phosphate) and one glyphosate metabolite (aminomethylphosphonic acid) were detected in these products [36]. Identification of other types of pesticides in edible insects was also achieved by Poma et al. [37] (e.g., azoxystrobine, cycloheximide and methoprene, among others). Another study examined 25 pesticides in multiple edible insects and found only isoproturon in *L. migratoria* (*n* = 1) [40]. Complementing these targeted approaches, a suspect screening study using high-resolution mass spectrometry identified 26 compounds across edible insect samples, with insecticides accounting for the largest share (42%), followed by herbicides (27%) [41]. The most abundant compound detected was trifloxystrobin, a broad-spectrum systemic fungicide, found in 16% of samples, while 14 samples contained no detectable compounds at all. Notably, samples originating from Europe showed fewer detectable compounds compared to those from Asia and Africa, with the highest number found in samples from Uganda. In contrast, a study screening 374 agrochemicals across wild-harvested and traditionally reared African edible insect species detected nine agrochemicals, comprising two insecticides (aminocarb and pymetrozine), three herbicides (atraton, methabenzthiazuron and metazachlor), and four fungicides (carboxin, fenpropimorph, fludioxonil and metalaxyl) [42]. While some species, including *Ruspolia differens* and adult *Oryctes* sp., were free from any detectable residues, others showed more concerning results: samples of *Pseudocreobotra ephippiata* reared on black soldier fly larval frass and plant compost, and *Rhynchophorus phoenicis* from oil palm, contained levels of atraton, methabenzthiazuron and metazachlor exceeding MRLs by up to 49-fold [42]. These results suggest that, although pesticide residues are present in some insect-based foods, the overall incidence of noncompliance is low, and most products meet established safety standards for general food products. However, it also highlights that wild-harvested or traditionally reared insects sourced from potentially contaminated environments may pose a greater compliance risk than those produced under controlled indoor farming conditions.

Although their application in insect farming is uncommon, veterinary drug residues may still be introduced through contaminated feed or environmental exposure if not properly managed. Insects reared on substrates such as manure may come into contact with veterinary drug residues, although this practice is currently prohibited in Europe [43]. Additionally, antibiotics and other veterinary drugs may be administered during insect farming to control disease outbreaks [28]. However, current EU legislation does not authorize the use of antibiotics in insect farming, aligning with the broader regulatory framework that restricts the use of antimicrobials in food-producing animals to combat antimicrobial resistance. Nevertheless, using antibiotics poses challenges, as it can influence insect development and survival and potentially contribute to the emergence of antibiotic-resistant pathogens [44].

Research on the presence of these residues in edible insects remains scarce. Hil et al. [44] demonstrated that the transfer of veterinary drug residues from a substrate to larvae can occur, although the concentrations were generally low (except for doxycycline). In a screening of 29 veterinary drugs, salicylic acid and metoprolol were detected in three of four insect species (lesser mealworm, black soldier fly and house cricket). Paracetamol and the mycotoxin zearalenone were detected in 37.5% and 19.75% of samples of four species of edible insects (n = 16), respectively [40]. In the United Kingdom, only nicarbazin (4,4′-dinitrocarbanilide) was detected in one sample of housefly, *Musca domestica*, grown on poultry manure [45]. Additionally, it has been shown that, in addition to uptake, veterinary drugs can also be metabolized and/or degraded during larval rearing [43].

While available studies suggest that residues of pesticides and veterinary drugs in insect-based foods are generally low, the rapid expansion of the sector and the use of diverse rearing substrates require continued vigilance. Notably, there are currently no insect-specific maximum limits for any pesticides and veterinary drugs, and the risk assessment process often relies on extrapolation from other food-producing animals (e.g., meat).

### 5.3. Persistent Organic Pollutants (POPs)

Persistent organic pollutants (POPs) are a group of synthetic organic compounds that are resistant to environmental degradation and can bioaccumulate through the food chain, posing significant health risks to both humans and wildlife [46]. The most relevant POPs in the context of insect-based foods include polychlorinated biphenyls (PCBs), polyaromatic hydrocarbons (PAHs), and dioxins. These compounds are of particular concern due to their persistence, toxicity, and potential to accumulate in lipophilic tissues [47].

Although their current use is prohibited (PCBs) or heavily restricted (PAHs and dioxins) under the Stockholm Convention, contamination can still occur through the environment, mainly in soils, sediment, and air [12]. Consequently, the concentration of these chemicals may substantially increase in the fat extracts of insects used for food [28]. A study of insect foods available on the Japanese market reported significant variations in PCB concentrations, ranging from 0.3 to 202 ng/g lipid weight (lw) in edible insects and 0.3 to 1.1 ng/g lw in insect-based foods (e.g., cricket-based pasta, cricket fruit bar). These values were lower than the Japanese reference values for PCBs in fish and meat (500 ng/g wet weight, ww) and eggs (300 ng/g ww) [48]. Similarly, Poma et al. [37] found that insects from Europe and Asia contained PCB levels generally comparable to other commonly consumed animal proteins and did not exceed the legal limits for animal-derived food in the country of purchase.

Charlton et al. [45] analyzed larvae of four fly species (*M. domestica*, *Calliphora vomitoria*, *Chrysomya* spp., and *H. illucens*) from various geographic regions and production systems, reporting that all samples contained dioxins and PAHs at concentrations well below the EU’s regulatory limits for animal feed. In this study, dioxins (expressed as World Health Organization toxic equivalent) ranged from 0.14 to 0.44 ng/kg (EU limit: 0.75 ng/kg). PAH4 (benzo[a]pyrene, benz[a]anthracene, benzo[b]fluoranthene, and chrysene) values in these samples ranged from 0.28 to 9.82 µg/kg, which fell within or below the established limits for various food products (range from 1 μg/kg in baby food to 35 μg/kg in smoked seafood). Similarly, Fels-Klerx et al. [49] investigated black soldier fly larvae reared on former foodstuffs and found that dioxin and PAHs concentrations were consistently low. Upper-bound dioxin values in these larvae ranged from 0.190 to 0.708 ng/kg, while PAH4 concentrations in these larvae were nearly zero.

Collectively, these findings indicate that, under current production practices and substrate choices, the risk of dioxin, PCB, and PAH contamination in insect-based foods is low, and the products are generally compliant with existing food and feed safety standards. From a regulatory perspective, the EU has established maximum levels for dioxins (for three of the four species permitted for human consumption, *T. molitor*, *A. domesticus* and *L. migratoria*) (Table 3) and PCBs in foods of animal origin; however, currently, there are only insect-specific limits for dioxins. As a result, risk assessments for insect-based foods typically rely on the standards set for conventional animal products.

### 5.4. Mycotoxins

Another group of contaminants that can be found in insect-based foods are mycotoxins. Mycotoxins are toxic secondary metabolites produced by certain fungi (mainly *Aspergillus* spp., *Fusarium* spp., and *Penicillium* spp.), commonly present in agricultural substrates such as fruits, rice, corn, and wheat, which are frequently used as feed for rearing edible insects [50,51]. Mycotoxins of concern include aflatoxins, fumonisins, zearalenone, ochratoxin, and deoxynivalenol, all of which are recognized for their carcinogenic, immunosuppressive, hepatotoxic, neurotoxic, and nephrotoxic effects in humans and animals [50].

Recent studies have shown that the accumulation of mycotoxins in edible insects is generally low, with most species demonstrating some ability to metabolize or degrade these compounds [50,52,53,54]. For example, in a study with *T. molitor*, larvae showed high tolerance for zearalenone-containing feed [54]. Additionally, it was also possible to observe that metabolic degradation and rapid excretion occur in these species [54]. Similarly, Camenzuli et al. [52] showed that α- and β-zearalenol (metabolites) were detected in the residue of *H. illucens* larvae, while only zearalenone was present in the initial feed. Furthermore, this study indicates that aflatoxin B1 and deoxynivalenol in this insect species may form additional metabolites, such as de-epoxynivalenol (a metabolite of deoxynivalenol which can be produced by bacteria in the gut) or conjugated forms of mycotoxins. It was also demonstrated that *T. molitor* had high elimination rates for ochratoxin A and fumonisin B1, with approximately 60% and 76% elimination rates, respectively [53]. In contrast, *H. illucens* larvae (Diptera) struggle to eliminate ochratoxin A, zearalenone, and deoxynivalenol, particularly at high substrate concentrations [50]. Despite this, there is no clear evidence that these toxins accumulate in larval tissues. Studies support the hypothesis that, since insects can metabolize and excrete these contaminants, their safety limits in feed intended for use as a substrate for growing insects may be higher than the current EC limits for feed for production animals. Furthermore, if insects are indeed capable of metabolizing and excreting mycotoxins, this could open an interesting avenue for the valorization of mycotoxin-contaminated cereal streams, which are currently difficult to remediate given the limited available decontamination methods. In this context, the use of contaminated substrates as insect feed could represent a novel biological strategy for mycotoxin mitigation, though further research is needed to fully characterize the metabolic pathways involved and to assess the safety of the resulting insect biomass.

Current EU legislation [55] has established a comprehensive regulatory framework for mycotoxins in conventional foodstuffs, setting maximum levels for a broad spectrum of toxins (aflatoxins, ochratoxin A, deoxynivalenol, zearalenone, fumonisins (B1 and B2), patulin, citrinin, ergot sclerotia and ergot alkaloids). Insect-based foods are regulated differently, with a narrower focus on specific mycotoxins, setting limit values only for aflatoxins, ochratoxin A, and deoxynivalenol (Table 4).

## 6. Microbiological Contaminants

The microbiological safety of insect-based foods is a critical aspect of their overall risk profile, as insects can harbor a diverse range of microorganisms. The farming environment plays a significant role in shaping the microbiota community in insects, modulating both intrinsic microorganisms and serving as a source for new ones [12].

In a study involving 52 samples of dried whole insects and insect powder collected for microbiological hazard testing, neither *Salmonella* spp. nor *E. coli* (>100 CFU/g) were detected [36]. These findings are consistent with more recent evidence from the US, where eight processed edible insect products, including crickets, mealworms, grasshoppers, silkworms, and diving beetles, were all found to be negative for *Salmonella*, with *Enterobacteriaceae* detected only in mole crickets and house cricket powder at low levels (<2.30 and <2.15 Log_10_ CFU/g, respectively) [56]. Similarly, a study on five types of frozen edible insects in Thailand found no *Salmonella*, and *Clostridium perfringens* and *Staphylococcus aureus* remained below the limit of quantification. However, *Enterobacteriaceae* and *E. coli* reached up to 5.05 and 2.70 Log_10_ CFU/g, respectively, in some samples, raising hygiene concerns [57]. In contrast, *Bacillus cereus*, *Aspergillus* spp. and *Penicillium* spp. were detected in dried and powdered insects from Europe and Asia [58]. This study also demonstrated that class I products (dried and powdered insects) exhibited significantly higher microbiological counts than the deep-fried and cooked ones (class II), suggesting that class I products should be thoroughly heated before consumption. In contrast, class II products were safer to consume directly, provided all precautions were taken as with more common foodstuffs. In fact, the application of processing methods such as boiling, blanching, steaming, roasting, and drying was effective at reducing microbial loads [59,60,61]. Garofalo et al. [62] also observed a great diversity and variation in bacteria among insects, noting relatively low counts of total mesophilic aerobes, *Enterobacteriaceae*, lactic acid bacteria, *Clostridium perfringens* spores, yeasts, and molds in all studied insect batches. Additionally, several gut-associated bacteria were identified through pyrosequencing, some of which may act as opportunistic pathogens in humans. This concern is further supported by the US study, in which whole genome sequencing of selected isolates identified 12 bacterial genera, with the majority belonging to *Bacillus*, and several isolates of the *B. cereus* group characterized as biovar Emeticus, a variant associated with emetic toxin production [56]. Krongdang et al. [57] similarly detected presumptive *B. cereus* ranging from <1.70 to 3.93 Log_10_ CFU/g across insect types, and amplicon sequencing revealed a diverse bacterial community dominated by Firmicutes and Proteobacteria, with notable phylum-level variation between insect species. In general, the three main bacterial genera/species associated with food safety problems in edible insects are *S. aureus*, *Clostridium* spp. (*Clostridium perfringens* and *Clostridium botulinum*) and the *B. cereus* group [63].

Current evidence on prions, foodborne viruses, and parasites in insects farmed for human consumption is still scarce but suggests a low risk [64]. This low risk was confirmed by Vandeweyer et al. [65] in industrially produced edible insects. No traces of the hepatitis A virus, hepatitis E virus, or norovirus genogroup II could be detected in any of the samples. Viral outbreaks are more likely to arise due to the introduction of pathogens during processing, which can be devastating for the productivity and quality of mass rearing systems [66].

In the EU, edible insects must comply with insect-specific microbiological criteria (Table 5). This constitutes the more defined criteria for edible insect regulation compared to all the contaminants previously mentioned. The regulation includes nine microbiological parameters but only four for mycotoxins, two for heavy metals, and only one for dioxins, illustrating how the criteria vary in number and focus across different contaminant types. However, more data on the microbiological quality of different insect species, the impact of various substrates and processing methods, and the potential for antibiotic resistance gene transfer within the insect microbiome are still needed.

As the sector expands, new pathogens may emerge, and developing insect-specific microbiological criteria will be vital for ensuring consumer safety. Establishing a preventive approach based on the Hazard Analysis and Critical Control Points system (HACCP) a widely recognized as a good practice throughout the edible insect industry. Many producers implement HACCP principles to systematically identify, assess, and control microbiological and chemical hazards throughout the production chain [67]. This structured approach enables the establishment of critical control points where contamination risks can be effectively minimized, thereby ensuring the safety and quality of insect-based foods.

## 7. Allergenic Risks

The allergenic potential of insect-based foods is a significant consideration for both food safety authorities and consumers, particularly as the market expands in Western countries. Typically, allergic reactions to insects occur due to occupational exposure, inhalation, direct contact, or bites and stings from insects, most often from Hymenoptera stings (such as bees and wasps) [68]. However, bites from blood-feeding insects, poisonous spines or hairs, and defensive secretions can also cause allergic reactions [12,68]. There have also been reports of allergic reactions to edible insects in various species, including silkworms, mealworms, caterpillars, *Bruchus lentis*, sago worms, locusts, grasshoppers, cicadas, bees, and *Clanis bilineata* [69,70,71,72,73]. The prevalence of insect-induced food allergies varies by region and population. Studies in Asian populations, where entomophagy is more prevalent, suggest that edible insects may contribute to 4.2–19.4% of food allergies and up to 18% of fatal food-induced anaphylaxis cases [74]. In Western countries, the risk remains poorly characterized, but could become more frequent in the future, raising important questions about hidden allergens and the need for clear labeling and risk communication.

These reactions can occur in both individuals with a history of atopy and those without, suggesting that insect consumption can result in both primary sensitization and cross-reactivity [75]. One of the primary concerns is the cross-reactivity between insect proteins and those found in crustaceans (such as shrimp and prawn) and house dust mites, as these groups share a close taxonomic relationship within the arthropod phylum [75,76]. The proteins tropomyosin and arginine kinase have been identified as major allergens in insects, and these are also well-known allergens in shellfish and mites. Consequently, individuals with existing allergies to crustaceans or dust mites may be at increased risk of allergic reactions when consuming insect-based foods [12]. Broekhoven et al. [77] reported in vitro cross-reactivity of immunoglobulin E from crustacean- and house dust mite-allergic patients to mealworm tropomyosin, α-amylase, hexamerin 1B precursor, and muscle myosin, respectively. Additionally, as insects can also be used as animal feed, Premrov Bajuk et al. [73] reported that dogs allergic to mites may also clinically express cross-reactivity with mealworm proteins.

Although thermal processing methods, such as blanching, pasteurization, and sterilization, are commonly used to ensure the microbiological safety of insect-based foods, they do not necessarily eliminate the allergenic potential of insect proteins [75]. In some cases, processing may even enhance the allergenic potency or alter the immunoreactivity of specific proteins, although the effects are highly variable and depend on the insect species and processing method [75]. Chemical or enzymatic hydrolysis has shown some promise in reducing allergenicity, but more research is needed to confirm its effectiveness across different insect species and food matrices [78].

Current risk assessments by EFSA and other regulatory bodies acknowledge the potential for both primary sensitization and cross-reactivity and recommend that insect-based foods carry appropriate allergen warnings, particularly for consumers with known shellfish or mite allergies [76]. Clear allergen labeling and the segregation of production lines are vital practical measures to manage allergenic risks in facilities that manufacture insect-based food. Transparent labeling ensures that consumers are fully informed of the presence of insect proteins, as recommended by the Food Information to Consumers (FIC) Regulation, and fulfil EU labeling requirements [79]. In parallel, many manufacturers adopt dedicated or thoroughly cleaned production lines to prevent cross-contamination between products containing insects and those without, mirroring the best practices established in diversified food factories. These combined strategies not only minimize allergen exposure but also promote consumer confidence and regulatory compliance in an evolving market for novel foods.

## 8. Challenges and Future Opportunities

Most studies reporting on the safety of edible insects highlight that chemical contaminants, especially heavy metals and persistent organic pollutants (POPs), are among the most critical issues, and thus require greater industry and regulatory focus. Unlike traditional livestock products, where microbiological risks dominate, insect food safety demands legislation and monitoring tailored to the specific chemical and biological hazards relevant to insect substrates and metabolism, areas that remain underrepresented in current monitoring frameworks. Therefore, regulatory frameworks should be adapted accordingly, ideally following prior monitoring and risk assessment approaches based on actual exposure and hazard data, to set meaningful contaminant limits.

Substrate quality primarily determines the chemical contaminant load—namely metals and POPs—and therefore must be closely controlled and monitored. In contrast, thermal processing and post-harvest treatments are essential for mitigating microbiological hazards, confirming that best practices like HACCP should extend not only to kitchen or processing areas but also to ingredient sourcing and feed controls. While substrate quality could be regulated, this requires an improved understanding of toxicokinetics and bioaccumulation.

Allergenic risks are substantial and necessitate not only rigorous labeling for consumers but also the separation of production lines and transparent traceability. As food allergies related to insects are commonly due to cross-reactivity with crustacean and mite proteins, research should continue to identify which insect species pose the highest risks and public campaigns may be needed to raise awareness. Moreover, understanding the current susceptibility of the population can provide necessary data for risk assessment.

Ultimately, edible insects offer significant nutritional and sustainability advantages, and available data suggest they pose relatively low contamination risks when properly farmed and processed. Continued development of insect-specific residue limits, monitoring, and consumer information will be crucial for safeguarding public health and unlocking the full potential of insects as safe and sustainable food sources. Current regulatory frameworks remain insufficiently adapted for insects and must become clearer and more comprehensive. They should support innovation and the sector’s safe growth without imposing unnecessary barriers. Such regulations must reflect the realities of global consumption while safeguarding public health.

Despite these positive findings, several knowledge gaps and regulatory challenges remain. The priority should be the establishment of contaminant limit standards specific to the most commercially relevant insect species covering a broader range of relevant contaminants. These species-specific thresholds should be developed in collaboration with regulatory authorities such as EFSA, using existing animal feed legislation as a baseline framework while accounting for insect-specific exposure routes. Advancing our understanding of the metabolic fate of contaminants in insects is equally important. Future research should prioritize toxicokinetic studies that characterize the absorption, distribution, biotransformation, and excretion of important contaminants across key insect species and developmental stages. To ensure cross-study comparability, these studies should employ standardized methodologies, including dose–response experiments under controlled rearing conditions. In parallel, long-term safety assessment frameworks must be developed. These should include multigenerational exposure studies to identify potential cumulative or delayed toxic effects, as well as substrate-controlled feeding trials that systematically vary contaminant loads. Bioaccumulation essays across successive life stages should build upon established ecotoxicological frameworks (e.g., OECD Test Guidelines 305, 317 and 321) together with bioconcentration factor calculations developed under EU REACH (Registration, Evaluation, Authorisation and Restriction of Chemicals) regulation, adapted to the biological and dietary context of edible insect species, for which no equivalent standardized protocols currently exist. The development of harmonized experimental design guidelines would further ensure that emerging data are both scientifically robust and directly applicable to risk assessment purposes and regulatory decision-making.

## 9. Conclusions

The growing interest in insect-based foods as sustainable and nutritious alternatives to conventional animal proteins has brought renewed attention to their safety profile, particularly regarding chemical and microbiological contaminants. This review highlights that, while the consumption of insect-based foods can be an exposure route to a range of potential hazards, including heavy metals/metalloids, pesticides, veterinary drugs, POPs, mycotoxins, microbiological contaminants, and allergens, current evidence suggests that, when produced under controlled conditions and in compliance with existing food safety regulations, the risks to consumers are generally low and comparable to those associated with other animal-derived foods. Moreover, the edible insect industry has made significant progress in controlling substrate quality, enhancing processing conditions, managing traceability, and improving hygiene protocols across production lines. In conclusion, insect-based foods present a promising avenue for diversifying the global protein supply, provided that food safety remains a key focus of industry development. Ongoing research, targeted monitoring, and regulatory adaptation will be essential to fully realize the benefits of edible insects while safeguarding public health.

## Figures and Tables

**Table 1:** Global frequency of consumption (%) of edible insect species per insect order. Adapted from Omuse et al. [13].

Common Names	Insect Order	Frequency of Consumption (%)
Beetles	Coleoptera	32.0
Bees, wasps, and ants	Hymenoptera	15.5
Butterflies	Lepidoptera	15.2
Grasshoppers, locusts, and crickets	Orthoptera	14.1
Cicadas, leafhoppers, planthoppers, scale insects, and true bugs	Hemipter	11.4
Termites	Isoptera	3.4
Dragonflies and damselflies	Odonata	2.4
Flies	Diptera	1.8
	All remaining taxa	4.2
Total		100

**Table 2:** Maximum limits of heavy metals in insect-based products in the European Union.

Species	Authorized Form	Pb (mg/kg)	Cd (mg/kg)	Regulation n.
*Tenebrio molitor*	Frozen	≤0.01	≤0.05	2022/169
	Dried or powder	≤0.075	≤0.1	2021/882
*Locusta migratoria*	Frozen, dried or powder	≤0.07	≤0.05	2021/1975
*Acheta domesticus*	Frozen, dried or powder	≤0.05	≤0.06 mg	2022/188
	Partially defatted powder	≤0.1	≤0.025	2023/5
*Alphitobius diaperinus*	Frozen, dried or powder	≤0.1	≤0.05	2023/58

**Table 3:** Maximum limits of toxins in insect-based products in the European Union.

Species	Authorized Form	Dioxins (UB, WHO-TEQ2005) (**) (pg/g fat)
*Tenebrio molitor*	Frozen	≤0.75
	Dried or powder	
*Locusta migratoria*	Frozen, dried or powder	≤1.20
*Acheta domesticus*	Frozen, dried or powder	≤1.25
	Partially defatted powder	
*Alphitobius diaperinus*	Frozen, dried or powder	NA

NA. Not available. (**) Upper-bound sum of polychlorinated dibenzo-para-dioxins (PCDDs)-polychlorinated dibenzofurans (PCDFs) and dioxin-like polychlorinated biphenyls (PCBs) expressed as World Health Organization toxic equivalent (using WHO-TEFs of 2005).

**Table 4:** Maximum limits of mycotoxins in insect-based products in the European Union.

Species	Authorized Form	Aflatoxins * (μg/kg)	Aflatoxin B1 (μg/kg)	Ochratoxin A (μg/kg)	Deoxynivalenol (μg/kg)	Regulation No.
*Tenebrio molitor*	Frozen	≤4	≤2	≤1	≤200	2022/169
	Dried or powder					2021/882
*Locusta migratoria*	Frozen, dried or powder	≤0.4	NA	≤1	≤200	2021/1975
*Acheta domesticus*	Frozen, dried or powder	≤4	≤2	≤1	≤200	2022/188
	Partially defatted powder					2023/5
*Alphitobius diaperinus*	Frozen, dried or powder	≤4	≤2	≤1	≤200	2023/58

NA. Not available. * Sum of aflatoxin B1, B2, G1, G2.

**Table 5:** Maximum limits of microbiology criteria in insect-based products in the European Union.

Species	Authorized Form	Total Aerobic Colony Count (CFU/g)	Yeasts and Molds (CFU/g)	*Escherichia coli* (CFU/g)	*Salmonella* spp.	Listeria Monocytogenes	Sulfite-Reducing Anaerobes (CFU/g)	*Bacillus cereus* (Presumptive) (CFU/g)	*Enterobacteriaceae* (Presumptive) (CFU/g)	Coagulase-Positive Staphylococci (CFU/g)	EU Regulations
*Tenebrio molitor*	Frozen	≤10^5^	≤100	≤50	Absence in 25 g	Absence in 25 g	≤30	≤100	≤100	≤100	2022/169
	Dried or powder								<10		2021/882
*Locusta migratoria*	Frozen, dried or powder	≤10^5^	≤100	≤50	Absence in 25 g	Absence in 25 g	≤30	≤100	≤100	≤100	2021/1975
*Acheta domesticus*	Frozen, dried or powder	≤10^5^	≤100	≤50	Absence in 25 g	Absence in 25 g	≤30	≤100	<100	≤100	2022/188
	Partially defatted powder						NA				2023/5
*Alphitobius diaperinus*	Frozen, dried or powder	≤10^5^	≤100	≤50	Absence in 25 g	Absence in 25 g	≤30	≤100	≤100	≤100	2023/58

NA. Not available.

## Data Availability

No new data were created or analyzed in this study.
